# Phylogeny and Molecular Characterisation of *PRNP* in Red-Tailed Phascogale (*Phascogale calura*)

**DOI:** 10.3390/brainsci15030250

**Published:** 2025-02-26

**Authors:** Krisel De Dios, Sachin Kumar, Ehsan Alvandi, Utpal Kumar Adhikari, Monique Amtoinette David, Mourad Tayebi

**Affiliations:** School of Medicine, Western Sydney University, Sydney, NSW 2560, Australiau.adhikari@westernsydney.edu.au (U.K.A.);

**Keywords:** marsupials, *Phascogale calura*, cellular prion protein, phylogenetic, protein stability

## Abstract

**Background/Objectives**: The normal cellular prion protein (PrP^C^) is a cell-surface glycoprotein, mainly localised in neurons of the central nervous system (CNS). The human *PRNP* gene encodes 253 amino acid residues of precursor PrP^C^. Several studies that investigated the role of *PRNP* and PrP^C^ in placental mammals, such as humans and mice, failed to reveal its exact function. **Methods**: In this study, we sequenced and characterised the *PRNP* gene and PrP^C^ of the marsupial, *P. calura*, as a strategy to gain molecular insights into its structure and physicochemical properties. Placentals are separated from marsupials by approximately 125 million years of independent evolution. **Results**: Standard Western blotting analysis of PrP^C^ phascogale displayed the typical un-, mono-, and di-glycosylated bands recognized in placentals. Furthermore, we showed that phascogale *PRNP* gene has two exons, similar to all the marsupials and placentals of the *PRNP* genes studied. Of note, the phascogale *PRNP* gene contained distinctive repeats in the PrP^C^ tail region comparable to the closely related Tasmanian devil (*Sarcophilus harrisii*) and more distantly related to the grey short-tailed opossum (*Monodelphis domestica*), common wombat (*Vombatus ursinus*), and Tammar wallaby (*Macropus eugenii*); however, its specific composition and numbers were different from placentals. Of importance, comparisons of the phascogale’s PrP^C^ physicochemical properties with other monotremes, marsupials, and placentals confirmed the Monotremata–Marsupialia–Placentalia evolutionary distance. We found that the protein instability index, a method used to predict the stability of a protein in vivo (Stable: <40; Instable >40), showed that the PrP^C^ of all marsupials tested, including phascogale, were highly stable compared with the birds, reptiles, amphibians, and fish that were shown to be highly unstable. However, the instability index predicted that all placental species, including human (*Homo sapiens*), mouse (*Mus musculus*), bank vole (*Myodes glareolus*), rhinoceros (*Rhinocerotidae*), dog (*Canis lupus familiaris*), flying fox (*Pteropus vampyrus*), whale (*Physeter catodon*), cattle (*Bos taurus*), and sheep (*Ovis aries*), were either slightly unstable or nearly unstable. Further, our analysis revealed that despite their predicted high PrP^C^ stability, *P. calura* exhibited substantial N-terminal disorder (53.76%), while species with highly unstable PrP^C^s based on their instability index, such as Danio rerio, Oryzias latipes, and Astyanax mexicanus, displayed even higher levels of N-terminal disorder (up to 75.84%). These findings highlight a discrepancy between overall predicted stability and N-terminal disorder, suggesting a potential compensatory role of disorder in modulating prion protein stability and function. **Conclusions**: These results suggest that the high stability of marsupial prion proteins indicates a vital role in maintaining protein homeostasis; however more work is warranted to further depict the exact function.

## 1. Introduction

The normal cellular prion protein (PrP^C^) is a cell-surface glycoprotein, mainly localised in the neurons of the central nervous system (CNS), with low levels of expression in peripheral organs [1]. The human *PRNP* is encoded to generate a precursor PrP^C^, which is 253 amino acid residues long [2]. The precursor PrP^C^ contains two primary domains, a disordered N-terminal region and a structured C-terminal domain, which are processed through two cleavage events [2,3]. The N-terminal region consists of an octarepeat and a hydrophobic region, and the C-terminal domain contains three α-helices, two β-sheets, and a signal sequence attached to a glycosylphosphatidylinositol (GPI) lipid anchor [3]. The cleavage event produces a mature PrP^C^ (208 amino acids in length) linked to the cell membrane via a C-terminal GPI lipid anchor [2,3,4]. PrP^C^ has an aggregated, β-sheet-rich counterpart, referred to as scrapie prion protein (PrP^Sc^) [5,6]. The conversion of PrP^C^ to PrP^Sc^ can occur via three different processes, including mutations of the *PRNP* gene that facilitate conversion of the normal to the diseased isoform, horizontal infection with biological materials containing PrP^Sc^, or spontaneous conversion of unknown origin [7,8]. The misfolding of PrP^C^ results in the development of multiple neurodegenerative diseases, such as kuru and Creutzfeldt–Jakob disease (CJD) in humans, scrapie of goats and sheep, and bovine spongiform encephalopathy in cattle [7,9,10]. PrP^Sc^ contains the same amino acid sequence as PrP^C^; however, it has different physicochemical features such as increased proportion of β-sheet structures, reduced solubility in non-ionic detergents, and partial resistance to protease digestion, such as proteinase K (PK) and thermolysin (THL) [11,12]. PK treatment digests the N-terminal end between residues 23 and 89 to create the truncated PrP^Sc^ isoform, termed PrP27-30 or PrP^res^, although it has been documented that PrP^Sc^ could be sensitive to PK digestion [13]. Prior to PK digestion, PrP^C^ normally exhibits a 3-band pattern, including mono-, di- and unglycosylated forms of PrP ranging between approximately 25 and 39 kDa [14]. In comparison, when PrP^Sc^ is treated with PK, a 3-band pattern profile between 18 and 30 kDa is displayed on Western blot [15]. In contrast, treatments with THL also digests PrP^C^ while preserving the full length PrP^Sc^ isoform, composed of PK-resistant and PK-sensitive PrP^Sc^ [11,16].

There are various types of PrPSc that have been classified based on their codon 129 status, molecular mass, fragment size when separated through SDS-PAGE, and degree of glycosylation [17]. Type 1, 2, and 6 of PrPSc are from sporadic CJD, type 3 is found in acquired CJD [17,18], and type 4 and 5 are associated with variant CJD [18,19,20,21]. Sporadic CJD cases are typically found to be homozygous for either methionine or valine at codon 129 in human PrP, which is associated with genetic susceptibility to prion disease [22,23,24]. Following protease digestion, the 3-band pattern of the types 1–6 range are approximately between 16 and 40 kDa [17,18,20]. Metal ion binding to PrPSc samples can alter the protein conformation and cause a band pattern shift [25]. There are discrepancies in the classification of PrPSc types due to the heterogeneity associated with the PrPSc samples used [26]. The classification of PrPSc types is based on glycoform profiles that have been reported as types 1–3 [17] and other types, types 1 and 2 [26]. Types 1–2 identified by Hill and colleagues (2003) equate to the single type PrPSc [26], and type 3 [17] corresponds to type 2 [26].

The function of the prion protein is not well understood, with multiple reports identifying a diverse repertoire of PrP^C^ functions, such as copper binding [27], T cell activation [28], and cell proliferation and differentiation [29,30]. A previous report from Premzl and colleagues (2005) described the *PRNP* gene structure of the Australian marsupial, Tammar wallaby following comparative genomics with a number of species. Interestingly, the authors showed that the Tammar wallaby displayed distinct repeats in the N terminal region [31], which has been linked to changes in conformational flexibility and phenotypic variability [32]. Comparison of the Tammar wallaby *PRNP* with other species also identified putative regulatory regions [33] and a possible interaction of PrP^C^ with MZF-1, MEF2, MyT1, Oct-1, and NFAT transcription factors, indicating its involvement in synaptic plasticity and signal transduction [31,34,35]. A previous study also compared the expression pattern of PrP^C^ in the CNS between the metatherian South American short-tailed opossum (*Monodelphis domestica*) and mouse [36]. This study found PrP^C^ expression varied between different regions of the CNS and during brain development between both species. This suggests differences in neurodevelopment between opossum and mouse and potentially extending to other species encoding PrP^C^. Additionally, these differences in PrP^C^ expression and distribution may also affect other features such as the susceptibility to prions [36].

In this study, we focused on the characterisation of PrP^C^ in *P. calura* [37], hereafter referred to as phascogale PrP^C^. The physicochemical properties of phascogale PrP^C^ were examined and compared with placentals, such as human and mouse, which are separated from marsupials by approximately 125 million years [38,39]. *P. calura* is an arboreal, insectivorous marsupial, belonging to the Dasyuridae [40]. The males of this species follow a semelparous reproduction pattern where total mortality among the adult males occurs after a single breeding period [41]. Previous molecular and histological studies on *P. calura* have primarily focused on investigating the cause of premature death in adult males after breeding [42,43], in the development of the *P. calura*’s immune tissues [44,45], and on various components of the immune system, such as the major histocompatibility complex and T-cell receptors [46,47]. This work is the first report describing the characterisation of PrP^C^ in *P. calura*. Our study shows a comparative analysis of the molecular and physicochemical properties of phascogale PrP^C^ with other species. The results of this comparative analysis confirm the evolutionary distance of the PrP^C^ sequence and stability in Monotremata, Marsupialia, and Placentalia. The comparison of the predicted instability index between species showed the highest stable form of PrP^C^ was found in *P. calura* and other marsupials. The presence of varying molecular and physicochemical properties in different species can provide insight into the evolution of the protein sequence and stability of PrP^C^. By extension, this might help with the understanding of the function(s) of PrP^C^.

## 2. Materials and Methods

### 2.1. Animal and Ethics

The 6 male and 2 female phascogale brain tissues used in this study are not subject to animal ethics guidelines. In accordance with the 3Rs, brain tissues were sourced from another independent study performed in accordance with the Australian Code for the Care and Use of Animals for Scientific Purposes and the New South Wales Animal Research Act and its Regulations. All protocols and standard operating procedures were approved by the WSU Animal Care and Ethics Committee and the NSW NPWS [46]. A full ethics statement under which the previous study was conducted can be found in [46].

### 2.2. Tissue Preparation and Homogenisation

Prior to removing of whole brains, *P. calura* carcasses were stored at −80 °C until further use. Whole brains were then removed from 6 males and 2 females, weighed, and homogenised in phosphate buffered saline (PBS) (Precellys 24 lysis and homogenisation machine, Bertin technologies, Montigny-le-Bretonneux, France) and aliquoted as 10% (*w*/*v*) stock solutions until further use.

### 2.3. Enzymatic Digestion with Proteinase K

Brain homogenates (10% *w*/*v*) were treated with proteinase K (PK). Samples were treated with 50 µg/mL PK, then placed on a shaker at 37 °C for 1 h.

### 2.4. Western Blotting Analysis

Brain tissues from phascogales were homogenised (10% *w*/*v* in PBS) using a Precellys^®^ 24 tissue homogeniser (Thermo Fisher Scientific, Scoresby, VIC, Australia), and centrifuged for 15 min at 1000× *g*. The supernatants were stored at −80 °C until use. Homogenates were diluted to 0.5% in complete Lysis-M buffer with Pefabloc SC plus protease inhibitors (Roche, Millers Point, NSW, Australia). Brain homogenates (10% *w*/*v*) were mixed with Laemmlie buffer (0.5%) (BioRad Laboratories, South Granville, NSW, Australia), then boiled at 95 °C for 5 min. Samples were loaded and separated by SDS-PAGE in 12% Tris-Glycine eXtended (TGX) gels (BioRad Laboratories Australia). Electrophoresis was conducted at 200 V for 5 min, and reduced to 100 V for 125 min. Next, proteins were transferred onto polyvynilidine fluoride (PVDF) membranes (BioRad Laboratories Australia) by electroblotting at 18 V for 2.5 h. Membranes were blocked with 5% skimmed milk on a shaker (42 rpm) for 1 h at RT. Membranes were then incubated overnight with the following anti-prion antibodies: SAF32 (1 µg/mL) or SAF70 (1 µg/mL). The membranes were then washed with phosphate buffer saline with 0.05% Tween 20 (PBST) prior to incubation with secondary anti-mouse IgG (1:3000) (Sigma Life Science, Melbourne, VIC, Australia). The membranes were then coated with ECL chemiluminescent substrate (Clarity^TM^ Western ECL substrate, BioRad Australia) for 5 min. Finally, membranes were imaged on iBright FL1000 (Thermo Fisher Scientific Australia).

### 2.5. Primer Design, DNA Extraction, PCR Amplification and Sequencing

Sequencing primers for phascogale *PRNP* were designed using the Tasmanian devil *PRNP* as a template (NCBI gene ID: 100927381). Using the Tasmanian devil *PRNP* as the DNA template sequence, the physical properties were determined using Oligocalc (version 3.27). Next, using the properties determined by Oligocalc, primers were generated by NCBI Primer-BLAST. Primer pairs generated by NCBI Primer-BLAST (https://www.ncbi.nlm.nih.gov/tools/primer-blast/ (accessed on 4 February 2022)) were validated using the in silico validation tool, Gene Runner (version 6.5.52).

DNA was extracted according to the manufacturer’s directions of the DNeasy Blood & Tissue Kit (Qiagen, Clayton, VIC, Australia). The concentration and purity of the extracted DNA was measured on a Qubit Flurometer (Thermo Fisher Scientific, Inc. Waltham, MA, USA). The extracted DNA was amplified in a PCR with a final reaction volume of 20 µL that included 10 µL of Invitrogen™ Platinum™ II Hot-Start Green PCR Master Mix (2×) (Thermo Fisher Scientific, Inc. Waltham, MA, USA), 5 pmol from each primer, and 20 ng of DNA. The PCR conditions were: 40 cycles at 94 °C for 20 s, followed by 60 °C for 20 s, and finally 68 °C for 30 s. FWD primer—AGATCAGCTACCATGGGAAAAATC—and REV primer—CTTGCAGACTGAAGGATTCCC—were used. PCR products were purified using Invitrogen PureLink^®^ PCR Purification Kit (Thermo Fisher Scientific, Inc. Waltham, MA, USA). Purified PCR products were sequenced in a Sanger sequencing reaction that included 27 ng of purified PCR production and 10 pmol of primer and yielded 801 base pairs. The sequencing was conducted by the Australian Genome Research Facility (AGRF) (Westmead Institute, Westmead, NSW, Australia).

### 2.6. Sequence and Comparative Alignment

The sequenced *PRNP* from phascogale was viewed using the SnapGene viewer program (from Insightful Science; available at https://www.snapgene.com/ (accessed on 4 February 2022)) and aligned to a sequence database using the NCBI BLAST tool. The ORF and protein sequence of *PRNP* were determined using NCBI ORF finder. The DNA sequence of phascogale *PRNP* was aligned using the Clustal 2.1 Alignment tool, then the comparative protein alignments were carried out using the Clustal Omega program (version 2.0) from UniProt (https://www.uniprot.org/ (accessed on 4 February 2022)).

### 2.7. Defining the Exon–Intron Structure of the Phascogale PRNP Gene

#### 2.7.1. Sequence Alignment for Exon Prediction

The genomic sequence of the phascogale *PRNP* gene, obtained through Sanger sequencing, was aligned with known annotated *PRNP* sequences from closely related marsupials (e.g., Tasmanian devil, grey short-tailed opossum) using Clustal Omega for multi-sequence alignment. Conserved regions corresponding to the coding exons were identified based on sequence similarity and evolutionary conservation.

#### 2.7.2. Open Reading Frame (ORF) Identification

The NCBI ORF Finder tool (https://www.ncbi.nlm.nih.gov/orffinder/ (accessed on 4 February 2022)) was used to identify the open reading frame (ORF) of the phascogale *PRNP* sequence. Exon regions were delineated by mapping the ORF to the sequenced genomic region. The ORF boundaries provided initial predictions of exon start and end positions.

#### 2.7.3. Genomic Annotation of Exon–Intron Boundaries

Exon–intron boundaries were predicted by comparing the genomic sequence with transcriptome data for the phascogale, as well as RNA sequences of related marsupial species (e.g., Tasmanian devil). BLAST (Basic Local Alignment Search Tool) was used to map known *PRNP* mRNA or cDNA sequences from other marsupials to the phascogale genomic sequence. Conserved donor (GT) and acceptor (AG) splice sites at the exon–intron junctions were identified using NetGene2 (https://services.healthtech.dtu.dk/service.php?NetGene2-2.42 (accessed on 4 February 2022)).

#### 2.7.4. Validation with Known Gene Structures

The exon–intron structure was cross-validated with the annotated *PRNP* gene structures in related species available in public databases, including Tasmanian devil (NCBI Gene ID: 100927381) and the grey short-tailed opossum (*Monodelphis domestica*, NCBI Gene ID: 554189). Comparison ensured that the predicted exon–intron organization of phascogale *PRNP* followed the typical two-exon structure found in marsupials and placentals.

#### 2.7.5. Functional Annotation and Final Confirmation

The predicted exon regions were confirmed by translating the sequence into amino acids and aligning the translated product with known PrP^C^ protein sequences from other marsupials. Any discrepancies were addressed by re-evaluating splice site predictions and transcript alignments.

### 2.8. Analysis of the Physicochemical Properties of Phascogale PrP^C^

We retrieved the prion protein sequences from the Uniprot database (https://www.uniprot.org/ (accessed on 4 February 2022)) for different species to analyse the physicochemical properties. We used phascogale male protein sequences and the sequences of multiple other species for comparative analysis using the expert protein analysis system ExPASy ProtParam server (https://web.expasy.org/protparam/ (accessed on 4 February 2022)) [48]. We considered the theoretical P^I^, the aliphatic index (AI), the half-life of protein, the instability index (II), and the grand average hydropathy (GRAVY) value for comparison purposes.

## 3. Results

### 3.1. Anti-PrP Monoclonal Antibodies Recognise Phascogale PrP^C^

**Figure 1:**
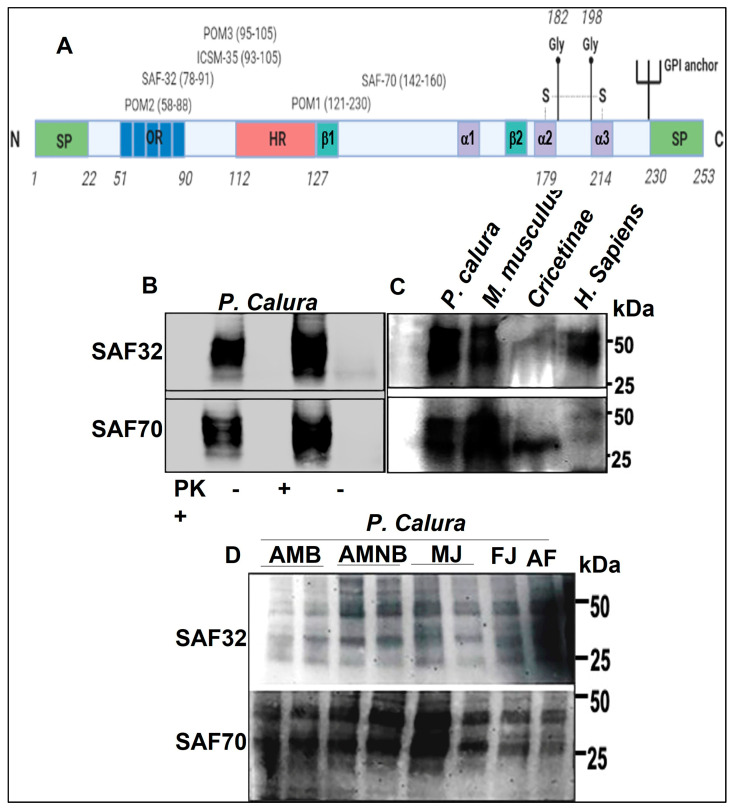
Detection of phascogale PrP^C^ with anti-PrP monoclonal antibodies. (**A**) IlFigurelustration of the prion protein and anti-PrP antibody epitope binding sites. SP = signal peptide; OR = octapeptide repeat region; HR = hydrophobic region; Gly = N-glycosylation sites; S-S; disulphide bridges. Antibodies: SAF32, SAF72, ICSM18. (**B**) Western blot analysis of 10% (*w*/*v*) *P. calura* brain homogenates treated with (+) or with no proteinase K (PK) using anti-PrP antibodies SAF32 and SAF70. (**C**) Western blot analysis of 10% (*w*/*v*) *P. calura*, mouse (*M. musculus*), hamster (Cricetinae), and human (*H. sapiens*) brain homogenates using anti-PrP antibodies SAF32 and SAF70. (**D**) Western blot analysis of 10% (*w*/*v*) *P. calura* brain homogenates derived from adult male breeders (AMB), adult male non-breeders (AMNB), male juvenile (MJ), a female juvenile (FJ), and an adult female (AF) using anti-PrP antibodies SAF32 and SAF70. Representative of 3 independent experiments.

A large number of anti-PrP antibodies have been produced that recognise both forms of PrP isoforms [49,50,51,52,53,54,55,56]. Scrapie-associated fibrils (SAF) derived from hamster were used as immunogen to generate an antibody response in both mice and rabbits. Although purified intact infectious SAF did not elicit detectable natural or experimental immune responses [53], a weakly measurable immunoreactive antibody reaction was evoked by formic acid-extracted SAF and SDS-solubilised SAF preparations, suggesting that treatment of SAF with detergent or denaturant somehow renders the antigen more immunogenic, perhaps by altering its conformation through a process of denaturation. For this study, we used SAF32 and SAF70, (Figure 1A) to characterise phascogale PrP^C^ using brain homogenates in Western blot (WB) to investigate the presence of the typical 3-band pattern (Figure 1B–D). Here, we demonstrate the presence of the 3–4 band pattern between 25–40 kDa (Figure 1B–D). To determine whether PrP^C^ derived from phascogale brain homogenates is susceptible to protease digestion, it was treated with proteinase K (PK) then analysed with WB using SAF32 or SAF70 anti-PrP mAbs (Figure 1B). Following treatment with PK, PrP^C^ was completely digested with 50 µg/mL for 1 h minimum at 37 °C (Figure 1B). In order to compare the molecular weight (MW) range of *P. calura* PrP^C^ with other well characterised mammalian PrP^C^s, 10% (*w*/*v*) *P. Calura* (Phascogale PrP^C^), *M. musculus* (Mouse PrP^C^), Cricetinae (Hamster PrP^C^) and *H. sapiens* (Human PrP^C^) brain homogenates were probed with either SAF32 or SAF70 following by Western blotting. Phascogale PrP^C^ displayed a similar MW with the other species tested, ranging between approximately 25 and 40 kDa (Figure 1C). Finally, we compared PrP^C^ derived from *P. calura*, including male and female juvenile, adult female, adult male (reproductively active and non-active). We showed that the sex, age, and reproductive status did not affect the MW of PrP^C^ (Figure 1D).

### 3.2. Sequence Homology of Phascogale PrP^C^ with Other Species

**Figure 2:**
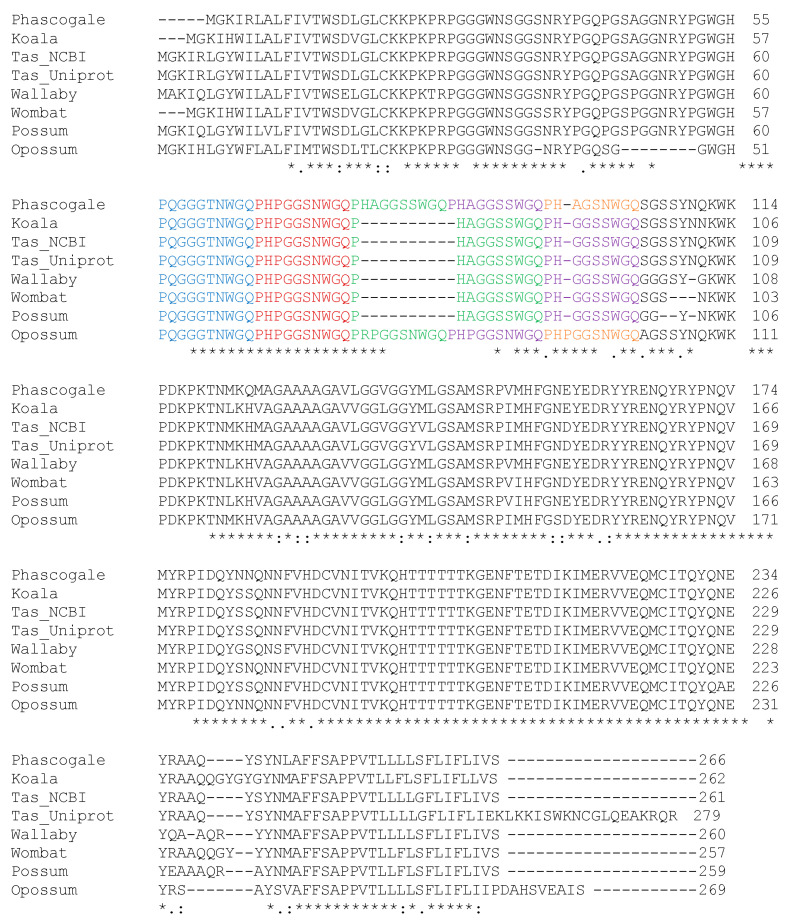
Multiple PrP^C^ sequence alignment of phascogale and other marsupials. Species including phascogale (*Phascogale calura*), koala (*Phascolarctos cinereus*), Tasmanian devil (*Sarcophilus harrisii*), Tammar wallaby (*Macropus eugenii*), common wombat (*Vombatus ursinus*), possum (*Trichosurus vulpecula*), and opossum (*Monodelphis domestica*). Fully conserved between all species: * (asterisk); Strongly conserved between species: : (colon); Weakly conserved between species: . (period); Repeats: #1 (blue), #2 (red), #3 (green), #4 (purple), #5 (orange).

**Figure 3:**
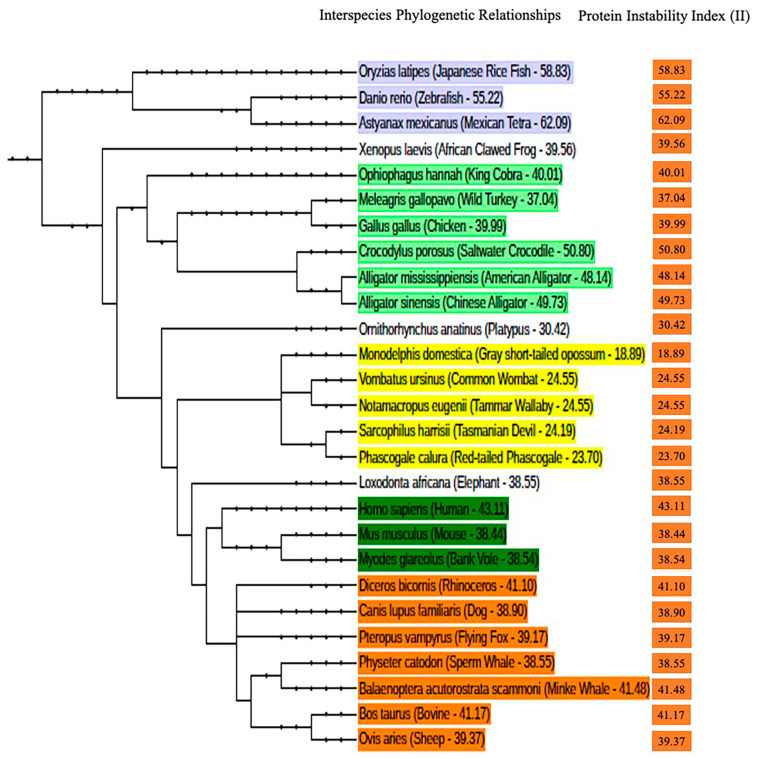
Interspecies phylogenetic relationship of marine, amphibian, reptilian, avian and mammalian species. The PrP^C^ instability index (II) is recorded next to each reported species. The colours in the phylogenetic tree represent different taxonomic groups of animals, helping to visually distinguish their evolutionary relationships: (i) **Blue**: Represents aquatic species, specifically fish and amphibians, such as the Japanese rice fish (*Oryzias latipes*), zebrafish (*Danio rerio*), and African clawed frog (*Xenopus laevis*). (ii) **Light-Green**: Represents reptiles and birds, including species like the king cobra (*Ophiophagus hannah*), saltwater crocodile (*Crocodylus porosus*), and wild turkey (*Meleagris gallopavo*). (iii) **Yellow**: Represents monotremes and marsupials, such as the platypus (*Ornithorhynchus anatinus*), common wombat (*Vombatus ursinus*), and Tasmanian devil (*Sarcophilus harrisii*). (iv) **Green**: Represents placental mammals, including humans (*Homo sapiens*), elephants (*Loxodonta africana*), and mice (*Mus musculus*). (v) **Orange**: Represents various mammalian species with higher protein instability index (II), such as dogs (*Canis lupus familiaris*), rhinos (*Diceros bicornis*), and whales (*Balaenoptera acutorostrata scammoni*).

To compare the level of sequence homology between *P. calura* PrP^C^ and a number of other species, DNA from two adult phascogale males were sequenced. The *P. calura* PrP^C^ sequence was submitted to GenBank and is available under the accession number. The DNA sequences of the phascogales were translated to amino acids and aligned with the published sequences derived from other marsupials (Figure 2) and non-marsupial species [57,58,59,60] using Clustal Omega [61]. Here, we show that the octapeptide repeat region of the *P. calura PRNP* and other marsupials is evolutionarily conserved with minor variations (Figure 3, shown in colour). Generally, the placentals studied contained an octapeptide repeat [62]; however, the marsupials displayed a mix of 4–5 nona- and deca-peptide repeats. The first (PQGGGTNWGQ) and second (PHPGGSNWGQ) repeats are identical among all marsupials [62]. The *P. calura PRNP* sequence displayed a much closer sequence similarity with Tasmanian devil and opossum [63], however, the alignment identified an even closer phylogenetic relationship with the *S. harrisii* (Figure 3). This shows a strong correlation between the PrP^C^ sequences similarity and close phylogeny amongst marsupials, suggesting a similar evolutionary split in protein sequence and species diversification of therians.

**Figure 4:**
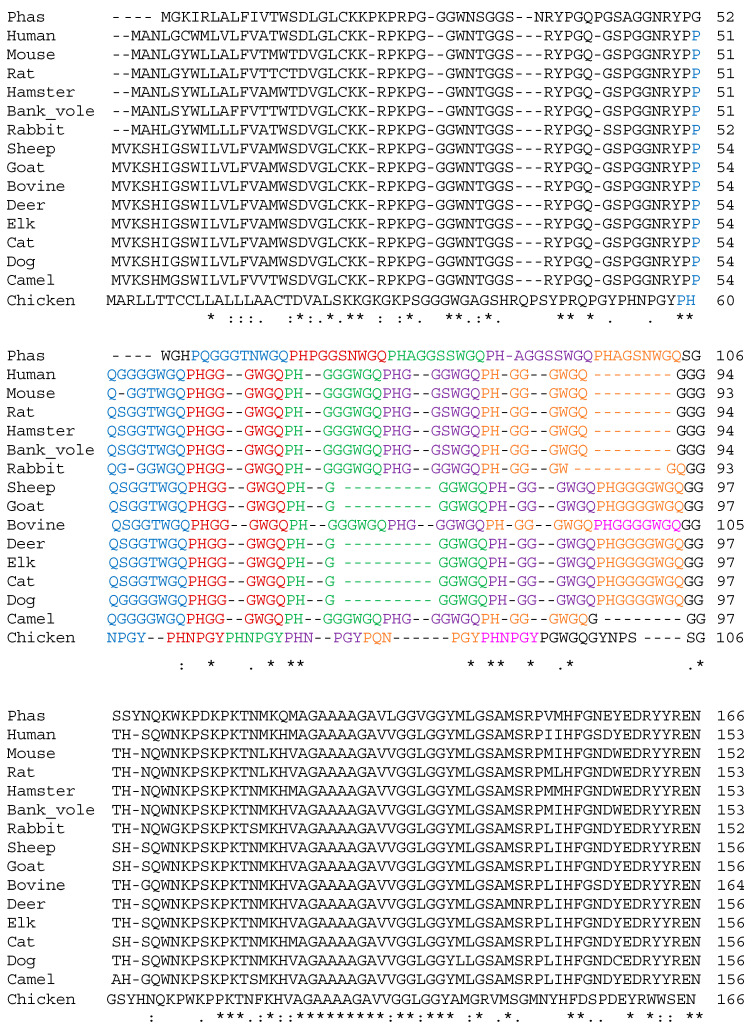

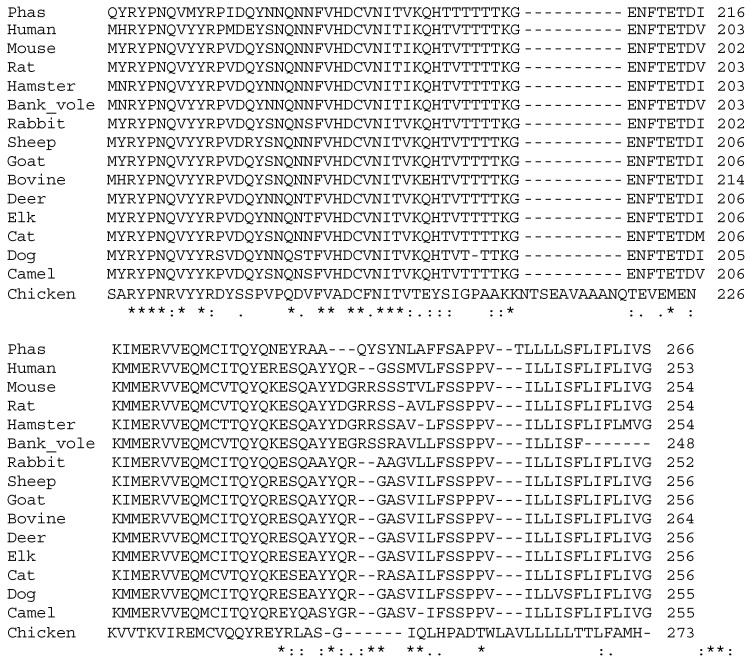
Multiple PrP^C^ sequence alignment of phascogale with mammalian and avian species. Species included phascogale (*Phascogale calura*), human (*Homo sapiens*), mouse (*Mus musculus*), rat (*Rattus norvegicus*), hamster (*Mesocricetus auratus*), bank vole (*Myodes glareolus*), rabbit (*Oryctolagus cuniculus*), sheep (*Ovis aries*), goat (*Capra hircus*), bovine (*Bos taurus*), deer (*Cervidae*), elk (*Cervus*), cat (*Felis catus*), dog (*Canis lupus familiaris*), camel (*Camelus dromedarius*), and chicken (*Gallus gallus domesticus*). Fully conserved between all species: * (asterisk); Strongly conserved between species: : (colon); Weakly conserved between species: . (period); Repeats: #1 (blue), #2 (red), #3 (green), #4 (purple), #5 (orange), #6 (pink).

Next, we performed multiple sequence alignments between PrP^C^ derived from *P. calura* and other mammalian and avian species. All the mammalian species, excluding cattle, contained five nona- and octa-peptide repeats similar to *P. calura* (Figure 4, shown in colour). Cattle shared the same number of repeats as chicken (Gallus gallus domesticus), however, the peptides and length of the repeat sequence differed. Cattle exhibited six nona- and octa-peptide repeats which had similar peptide sequences to the other mammalian species. In contrast, the sequence alignment showed that chicken PrP^C^ had six peptide sequences which were present as short hexa-repeats (Figure 4). When compared with other species, excluding marsupials, *P. calura* displayed the highest sequence similarity with chicken. The close relationship between *P. calura* and chicken is further supported by their closer phylogenetic relationship compared with other mammals (Figure 3). Following *P. calura*, in descending order of sequence similarity, a new tree node was created for each species, starting with rabbit (Oryctolagus cuniculus) and then camel (Camelus dromedarius), dog, cattle, cat (Felis catus), elk (Cervus), deer (Cervidae), and finally sheep and goat (Capra hircus) (Figure S1). This group of animals, excluding rabbit, camel and cattle, formed a separate phylogeny tree branch. Another phylogeny tree branch of hamster (Mesocricetus auratus), bank vole, mouse, and rat (Rattus norvegicus) was also formed, with the sequence similarity depicting it in a cluster with the human PrP^C^ sequence. The repeat sequences found between placental and marsupial mammals differ, each containing species-specific repeat sequences (Table 1).

### 3.3. A Comparative Analysis on the Physicochemical Properties of Phascogale PrP^C^

The physicochemical properties (PCP) of two male *P. calura* PrP^C^ protein sequences were predicted through in silico analysis, one with an N72N and the other with an N72S. Of note, both forms were included in the Western blotting analysis (Figure 1B–D). Along with the *P. calura*’s, the PCP of 27 other species including the human and mouse PrP^C^ sequences were also predicted (Table 2). The selected protein sequences showed that the length of most PrP^C^s is ~260 amino acids except zebrafish prion protein 1 (Danio rerio—567 and 606 amino acids), Japanese rice fish (Oryzias latipes—420 amino acids) and Mexican tetra (Astyanax mexicanus—594 amino acids). Similarly, *P. calura* PrP^C^ was found to be 266 amino acids in length. The isoelectric point, commonly known as the theoretical pI (pI), provides information about the acidic or basic nature of the protein and the average theoretical pI ranged between 8.74 and 9.62 for the selected species and 9.44 for both N72N and N72S phascogale. The instability index (II), which provides an estimate of the stability of proteins in a test tube, was also assessed [64]. As reported by Rogers and colleagues [65], proteins with an in vivo half-life of >16 h or <5 h were considered stable or unstable, respectively. The authors based their analysis on the frequency of occurrence of specific amino acids in the stable and unstable protein classes studied in vitro, then predicted stable and unstable amino acid ‘hot spots’ in proteins using the Protein Sequence Database of the PIR (Release 12.0). For instance, and in the case of unstable proteins, the presence of Met(M), Gln(Q), Pro(P), Glu(E), and Ser(S) was relatively high. Moreover, Guruprasad and colleagues [66] also demonstrated that the in vivo instability of proteins is determined by the order of certain amino acids in their sequence. If the II is <40, this indicates that the assessed protein is stable. Here, we predicted 12 unstable PrP proteins that displayed a value >40, including human (43.11), cattle (41.17), common minke whale (Balaenoptera acutorostrata scammoni—41.84), black rhinoceros (Diceros bicornis—41.10), king cobra (Ophiophagus hannah—40.01), Mexican tetra (62.09), Japanese rice fish (58.83), and zebrafish (55.22). Of importance, the II of both N72N and N72S *P. calura* PrP^C^ had values of 23.70 and 22.59, respectively, indicating that *P. calura* PrP is highly stable, similar to other marsupials, such as Tasmanian devil (24.19), wombat (24.55), and Tammar wallaby (24.55). Overall, the protein instability index of PrP^C^ differs in multiple species, which predominantly appears to have evolved into a more unstable form in many different species. Several classes of species have developed into separate phylogenetic tree clusters. Each cluster shows they have evolved similar protein instability indexes relative to other classes of species in the other tree clusters (Figure 3, shown in colour). Of particular importance, despite their predicted high PrP^C^ stability, both N72N and N72S *P. calura* exhibited a relatively high level of disorder in their N-terminal region (53.76% for both variants). This suggests that N-terminal disorder is not necessarily correlated with overall PrP^C^ stability, highlighting a possible functional or structural role independent of the intrinsic stability of the protein.

Moreover, species predicted to have highly unstable PrP^C^ based on their instability index (II), such as *Danio rerio* (DANRE Prion protein 1), *Oryzias latipes*, and *Astyanax mexicanus*, displayed even higher levels of N-terminal disorder (70.63%, 75.84%, 60.48%, and 66.67%, respectively). This suggests a potential compensatory mechanism, where increased N-terminal disorder may counterbalance the instability of the protein, possibly by influencing its conformational plasticity, interaction with binding partners, or susceptibility to misfolding.

We then conducted a comparison of the PrP^C^ phascogale and PrP^C^ of the other marsupial and mammalian species through analysis of its structural motifs. All the structural PROMOTIF information (sheet, beta hairpin, beta bulge, strands, helices, helix–helix interacts, beta-turn, gamma turn, and disulphides) are represented in Table 3. The PROMOTIF analysis revealed that there are structural differences mainly in beta-turn, gamma turn, strand, and helices between the homozygote phascogale and heterozygote phascogale. The structural differences between the phascogale and other marsupial and mammalian PrP^C^ were found mainly in the helices, beta-turn, and gamma turn level. The highest number of beta-turns were seen in the Tasmanian devil and the phascogales which exhibited 69 and 63 beta-turns. In contrast, there were several commonalities shown in the structural motif with similar numbers of beta sheet, hairpin, strand, and disulphide bonds between the heterozygote phascogale and other mammalian and marsupial species [67].

## 4. Discussion

To further explore the phylogenetic relationship in different mammals, we carried out a detailed analysis of the molecular and physiochemical properties of PrP^C^ in the marsupial red-tailed phascogale (*P. calura*). Here, we compared PrP^C^ properties between the marsupial phascogale and a number of phylogenetically distant placentals and monotremes. This study confirmed that *P. calura* PrP^C^ possesses similar molecular and physicochemical properties compared to other marsupials studied [68]. In this study, the sequence similarity and the number of repeats shared between Tammar wallaby, possum, koala, and wombat show a close *PRNP* phylogenetic relationship. This close relationship is likely attributed to belonging to the same marsupial Diprotodontia order [69]. Collectively, the various marsupial species are phylogenetically closer than other mammalian species [63]. *P. calura* PrP^C^ displayed the typical 3-band pattern profile which represents the mono-, di-, and unglycosylated forms of PrP^C^, albeit its SDS-PAGE migration profile ranged between 25 and 75 kDa compared to the typical 25–39 kDa range [14]. PrP^C^ in phascogale and other marsupials showed striking differences in their amino acid sequence, and structural and physicochemical properties when compared with placentals and monotremes. Aside from differing methods of embryonic development [70], other evolutionary differences have been highlighted among the mammalian subclasses, including genomic imprinting [71,72] and mitochondrial genome [73]. The chicken belongs to the Aves vertebrate class and its genome differs from mammalian vertebrates [74]; thus its PrP^C^ sequence is unique to its class and does not share sequence homology with either placental or marsupial mammals.

Of importance, we established that the evolutionary relationship of PrP^C^ seems to be intimately linked to its stability, progressing from a very low, to moderate and finally to a highly stable PrP^C^, as measured by the II [75,76]. This high variability of the PrP^C^ II appears to be associated with the evolutionary split; with very high II in fish (55 to 62), moderately instable in reptiles (40–50), moderately stable/instable (38 to 43) in placental mammals, such as human and mouse, and highly stable in marsupials where the II ranged between 19 to 24 [77]. Of note, the instability index of proteins was shown to be inversely proportional to the protein’s half-life [78]. Idicula-Thomas and Balaji (2005) have shown that proteins with in vivo half-life <5 h had a II >40 while proteins with half-life >16 h had a II <40 [65,66]. The inverse relationship between half-life and instability index was first shown by Guruprasad and colleagues (1990), although, with the exception of the highly stable RNase A that has a long in vivo half-life of 61 h despite a very high II value (=52.2). This example highlights that there might be other factors involved in protein stability; for RNAse A, it was speculated that in this case the presence of four disulphide bonds confers high stability [79]. PrP^C^ is composed of a flexible N-terminal and a globular C-terminal domain, consisting of three α-helices and two short β-sheets. α-helices 2 and 3 are linked by a disulfide bond at position ~178 and 213 in human PrP^C^ [3]. Furthermore, the in vivo half-life of PrP^C^ was approximately 18 h in a mouse model prion disease [80]. It was previously reported that reducing the disulfide bond led to α-helice 1 shift, β-sheet elongation, and de-stabilisation of human recombinant PrP^C^ [81]. Despite the large disparity of the II of PrP^C^ in placentals, marsupials, and monotremes studied here, the predicted in vivo half-life (30 h) was not affected and remained high except for the African bush elephant (Loxodonta africana) and platypus (Ornithorhynchus anatinus) with a half-life/II of 1.9 h/38.55 and 3.5 h/30.42, respectively. In contrast, *P. calura* PrP^C^, 266 amino acid residues long, has its cysteines located on residues 192 and 227. Taken together, these findings underscore the complex interplay between PrP^C^ stability and structural disorder, indicating that N-terminal disorder may serve a distinct functional role beyond mere stability determinants. This could have implications for prion protein evolution, function, and susceptibility to misfolding-related diseases.

Interestingly, placentals and monotremes which predominantly displayed a higher number of five and six repeats, also had higher II values. The increase in repeat copies has been observed to be associated with a higher susceptibility to prions (TSEs) [82]. This correlation between a higher susceptibility to TSEs and increased repeats could be linked to high protein instability. However, the protein stability and additional repeats may not be the only factors conferring susceptibility to prion disease. Dogs and rabbits exhibit five repeats, with the dog closely approaching an unstable protein index, but being resistant to prion disease [83,84].

The sequence of some regions of the PrP^C^ sequence are highly conserved in evolution [85], emphasizing a key role for the protein. Moreover, PrP^C^ presence in *P. calura*, a species separated from placentals by approximately 125 million years of independent evolution, in a highly stable form [86], further strengthen the proposition of the importance of this protein in biological systems. Overall, the characterisation of PrP^C^ in the marsupial, red-tailed phascogale has provided insights into the sequence evolution and diverse stability forms of PrP^C^. The consideration of the commonalities and variations established in PrP^C^ between multiple species during evolution can help clarify the main functional significance of this highly conserved protein.

## 5. Conclusions

In conclusion, we highlight the importance of studying the *P. calura* and other marsupial sequences to help further elucidate the disease related *PRNP* genes in other mammals. Our comparison of the *P. calura PRNP* with eutherian sequences identified a number of physicochemical properties and specific repeats in the flexible region unique to marsupials which might confer disease resistance. However, sequence conservation between marsupials and eutherians indicates that *P. calura* might display similar disease susceptibility [87]. The semelparous nature of *P. calura* is unlikely to play a role in disease resistance/susceptibility as this is largely dictated by the *PRNP* primary sequence and conformation [88]. Finally, this study warrants further investigation of the biological function(s) of PrP^C^ in marsupials.

## Figures and Tables

**Table 1:** Common and unique repeat sequences between mammalian and avian species. Common repeats are sequences identified in more than one species. Unique repeats are sequences that are only found in a single animal.

**Common Repeats**			
**Placental**		**Marsupial**	
PQGGGGWGQ PHGGGWGQ PHGGSWGQ PQSGGTWGQ PHGGGGWGQ		PQGGGTNWGQ PHPGGSNWGQ PHAGGSWGQ PHGGSSWGQ	
**Unique Repeats**			
**Placental**	**Marsupial**		**Avian**
PQGGGWGQ (rabbit) PQGGTWGQ (mouse)	PHAGSNWGQ (phascogale) PRPGGSNWGQ (opossum)		PHNPGY (chicken) PQNPGY (chicken)

**Table 2:** Physicochemical properties of phascogale PrP^C^. The number of residues, MW, net charge, IUR, and instability index. Phascogale (NN): N72N. Phascogale (NS): N72S.

Species	Number of Residues	MW	Net Charge (Z) at pH 7.4	Instability Index	Intrinsic Unstructured Regions (IUR)				Domain Position	
					No. of Disordered Regions	No. of Disordered Residues	Overall Disordered (%)	Disordered Segment	N-Terminal Domain (Disordered)	C-Terminal Domain
*Male breeder_N (HA)*	266	29,259.70	+8.201	22.59	2	143	53.76	19–145; 211–226	19–144	145–266
*Male breeder_S (HG)*	266	29,232.68	+8.201	23.70	2	143	53.76	19–145; 211–226	19–144	145–266
*Opossum*	269	29,757.34	+5.297	19.89	4	139	51.67	1–2; 25–140; 208–221; 263–269	25–141	142–269
*Tasmanian devil*	279	31,064.02	+13.063	24.19	4	139	49.82	2–2; 25–138; 206–221; 272–279	25–139	140–279
*Common wombat*	257	28,406.81	+7.246	24.55	2	128	49.81	22–132; 200–216	22–133	134–257
*Tammar wallaby*	260	28,605.13	+7.199	24.55	3	126	48.46	2–2; 26–138; 205–216	25–138	139–260
*Bank vole*	248	27,258.44	+7.259	38.54	2	122	49.19	23–130; 198–211	23–131	132–248
*Mouse*	254	27,977.41	+7.254	38.44	2	121	47.64	23–129; 197–210	23–130	131–254
*Dog*	257	27,779.31	+7.251	38.90	2	124	48.25	25–134; 202–215	25–135	136–257
*Sheep*	256	27,915.46	+9.299	39.37	2	127	49.61	25–133; 202–219	25–134	135–256
*Cow*	264	28,614.17	+7.395	41.17	2	138	52.27	25–141; 207–227	25–142	143–264
*Human*	253	27,661.17	+5.193	43.11	2	129	50.99	23–130; 198–218	23–131	132–253
*Gallus gallus doesticus*	273	29,908.68	+6.321	39.99	5	126	46.15	1–2; 24–102; 105–112; 133–143; 211–236	25–144	145–273
*Loxodonta africana*	253	27,517.91	+7.091	38.55	2	127	50.20	22–131; 199–215	22–132	133–253
*Meleagris gallopavo*	271	29,770.51	+7.276	37.04	5	128	47.23	1–2; 24–101; 104–111; 132–143; 208–235	25–143	144–271
*Xenopus laevis*	217	24,444.79	+8.711	39.56	3	91	41.94	1–5; 21–98; 174–181	24–99	100–217
*Alligator sinensis*	248	27,832.28	+6.338	49.73	3	120	48.39	23–90; 95–130; 196–211	25–131	132–248
*Balaenoptera acutorostrata scammoni*	257	27,945.52	+8.179	41.84	2	124	48.25	25–133; 202–216	25–134	135–257
*Physeter macrocephalus*	259	28,316.90	+8.341	38.55	2	126	48.65	25–135; 204–218	25–136	137–259
*Diceros bicornis*	248	26,915.10	+7.309	41.10	3	130	52.42	25–133; 201–220; 245–245	25–134	135–248
*Pteropus vampyrus*	264	28,402.83	+8.345	39.17	2	138		25–140; 209–230	25–142	143–264
*Alligator mississippiensis*	298	33,105.67	+9.636	48.14	3	124	41.61	73–140; 145–180; 242–261	74–160	161–298
*Crocodylus porosus*	260	29,048.61	+6.282	50.80	2	141	54.23	23–142; 202–222	25–143	144–260
*Ophiophagus hannah*	242	26,512.27	+11.139	40.01	5	138	57.02	1–5; 21–88; 90–126; 185–204; 219–226	25–129	130–242
*Ornithorhynchus anatinus*	215	23,331.96	+4.129	30.42	2	111	51.63	27–131; 196–201	26–124	125–215
*Danio rerio (DANRE Prion protein 1)*	606	62,806.21	+19.995	44.44	5	428	70.63	25–335; 427–448; 466–527; 535–541; 560–584	25–365	366–606
*Danio rerio (DANRE Prion protein 1)*	567	58,793.85	+4.481	55.22	6	430	75.84	25–255; 276–284; 310–311; 348–384; 400–520; 522–551	20–315	316–567
*Oryzias latipes*	420	45,208.90	+11.826	58.83	5	254	60.48	1–5; 22–199; 218–232; 320–363; 383–394	27–266	267–420
*Astyanax mexicanus*	594	62,139.06	+4.289	62.09	6	396	66.67	40–252; 290–299; 324–325; 362–396; 418–526; 547–573	35–329	330–594

**Table 3:** Structural properties of phascogale PrP^C^. The number of beta sheets, beta hairpin, beta bulge, beta stands, helices, helix–helix interacts, beta-turn, gamma-turn, disulphide structures are listed. *P. calura* N72S displays 2 beta sheets while other species analysed, including *P. calura* N72N, have 1 or no beta sheets.

Species	Beta Sheet	Beta Hairpin	Beta Bulge	Beta Strands	Helices	Helix-Helix Interacts	Beta-Turn	Gamma-Turn	Disulphides
*Phasogale (NN)*	1	1	0	2	5	3	69	11	1
*Phasogale (NS)*	2	2	0	4	7	3	63	17	1
*Human*	1	1	0	2	6	4	21	10	0
*Mouse*	1	1	0	2	5	2	58	13	1
*Sheep*	0	0	0	0	4	2	49	12	1
*Cow*	1	1	1	2	5	3	48	17	1
*Dog*	1	1	0	2	7	4	51	15	1
*Bank vole*	1	1	1	2	5	2	40	19	1
*Opossum*	1	1	1	2	4	3	55	13	1
*Tamanian devil*	1	1	0	2	8	3	79	17	1
*Wombat*	1	1	1	2	6	2	58	19	1
*Wallaby*	1	1	1	2	5	5	47	18	1

## Data Availability

The original contributions presented in this study are included in the article and Supplementary Material. Further inquiries can be directed to the corresponding author.

## Supplementary Materials

The following supporting information can be downloaded at: https://www.mdpi.com/article/10.3390/brainsci15030250/s1, Figure S1: Simplified tree diagrams of the sequence similarity of phascogale with an avian and other mammalian species.
