# Supplementary material for: Phylogeny and Molecular Characterisation of PRNP in Red-Tailed Phascogale (Phascogale calura)
Source: Brain Sci. 2025 Feb 26;15(3):250. doi: 10.3390/brainsci15030250 (PMC11940036; doi:10.3390/brainsci15030250)
Supplement: Supplementary file 1 [file brainsci-15-00250-s001.zip › brainsci-3391999-supplementary.pdf]

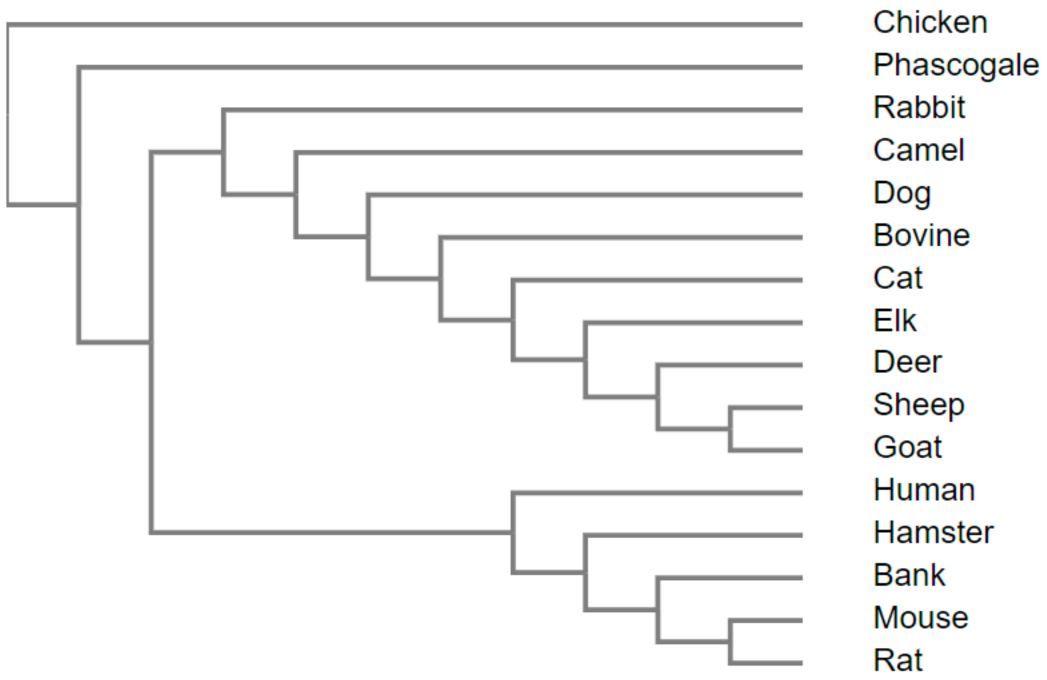

Figure S1. Simplified tree diagrams of the sequence similarity of phascogale with an avian and other mammalian species. Species: phascogale (*Phascogale calura*), human (*Homo sapiens*), mouse (*Mus musculus*), rat (*Rattus norvegicus*), hamster (*Mesocricetus auratus*), bank vole (*Myodes glareolus*), rabbit (Oryctolagus cuniculus), sheep (Ovis aries), goat (*Capra hircus*), bovine (*Bos taurus*), deer (*Cervidae*), elk (*Cervus*), cat (*Felis catus*), dog (*Canis lupus familiaris*), camel (*Camelus dromedarius*), and chicken (*Gallus gallus domesticus*).
